# Supplementary material for: Oxidative Stress and Antioxidant Defense in the Heart, Liver, and Kidney of Bat Species with Different Feeding Habits
Source: Int J Mol Sci. 2023 Nov 15;24(22):16369. doi: 10.3390/ijms242216369 (PMC10671108; doi:10.3390/ijms242216369)
Supplement: Supplementary file 1 [file ijms-24-16369-s001.zip › pharmaceuticals-2627169-Supplementary Materials.pdf]

International Journal of *Molecular Sciences*

MDPI

S1. Biochemical Analysis

S1.1. Oxidative damage

To assess protein damage, levels of carbonyl groups were determined using 2,4-dinitrophenyl hydrazine (DNPH) and measured at a wavelength of 370 nm [1]. The quantification of carbonyl levels was expressed as nmol of carbonyl per milligram of protein.

For the measurement of lipid peroxidation as an indicator, malondialdehyde (MDA) levels were determined by high-performance liquid chromatography (HPLC) using a reversed-phase column (SUPELCOSIL™ LC-18-DB HPLC column; 15 cm × 4.6 mm, 5 μm). The mobile phase consisted of a mixture of 30 mmol/L monobasic potassium phosphate (pH 3.6) and methanol (9:1, v/v) with a flow rate of 1 mL/min. Samples were injected in a volume of 25 μL, and the absorbance of the column effluent was monitored at 254 nm. The retention time of MDA under these conditions was 5.6 min [2]. MDA levels were expressed as nmol of MDA per milligram of protein.

S1.2. Enzymatic activity

The SOD activity was determined using the RanSOD kit from Randox, County Antrim, UK. Measurement of fumarase activity involved the conversion of fumarate to malate, with detection performed at 240 nm [3]. The enzymatic kinetics of glutathione peroxidase (GPx) were evaluated using the Ransel Kit from Randox, County Antrim, UK. The GST antioxidant assay measured the formation of S-(2,4-dinitrophenyl)-glutathione through the enzymatic activity of GST via the conjugation of 1-chloro-2,4-dinitrobenzene (CDNB) and glutathione (GSH). The measurement of GST activity was carried out by determining the absorbance at a wavelength of 340 nm [4]. All enzyme activities were quantified as U/mg of protein.

The consumption of H_2O_2 was evaluated with a protocol adapted for microplates [1]. 30% H_2O_2 was diluted in 10 mL of sodium phosphate buffer (50 mmol/L, pH 7), and added into the sample to trigger the reaction measuring the rate of H_2O_2 consumption via absorbance at 240 nm. H_2O_2 consumption was reported because there are multiple H_2O_2 detoxification mechanisms (mainly CAT and peroxiredoxins), and the test is not specific for any of them [5]. The activity was expressed as μmol of H_2O_2 consumption/min/mg of protein.

All assays were independently performed in triplicate.

S1.3. Non-enzymatic antioxidants

To indirectly measure the levels of nitric oxide, a modified version of the Griess test was employed to determine the total amounts of nitrate and nitrite (NO2 and NO3) [6]. Indirect nitric oxide levels were expressed as nmol of NO_2 and NO_3 /mg protein.

GSH levels were evaluated, preparing a mixture with a solution: 5,5-dithiobis (2-nitrobenzoic acid, DTNB), glutathione reductase (GR, 250 U/mL), and NADPH to convert GSSG into GSH and trigger GSH. GSSG levels were measured and treated with 2-vinylpyridine which reacts covalently with GSH and was measured at 412 nm [7]. Results are presented as GSSG/GSH ratio.

S1.4. Statistical Analysis

We conducted statistical analyses using the software PAST (version 4.12) to explore the relationships between antioxidant enzyme levels, oxidative stress marker levels and feeding guild in four bat species. Specifically, we performed a One-way PERMANOVA, Principal Component Analysis (PCA), and correlation analysis, as follows.

To test significant differences in the mean levels of each antioxidant and oxidative stress marker among the four bat species, we conducted One-way PERMANOVA. We first checked for normality using the Shapiro-Wilk test and verified the homogeneity of

variances using Levene's test. Since these assumptions were not met (< 0.05), we performed the non-parametric test One-way PERMANOVA followed by a Bonferroni-corrected PERMANOVA pairwise comparison to compare pairwise differences between the bat species.

To explore patterns of variation among the variables, we conducted PCA. Before PCA, we performed a transformation method to eliminate the effects of differences in scale, which involved subtracting the mean of each variable from its values. We then conducted PCA on the covariance matrix of the normalized variables.

We also conducted correlation analysis to explore the relationships between the different antioxidant enzymes and oxidative stress marker levels. We calculated Spearman correlation coefficients for pairs of variables and tested their significance using two-tailed tests.

All statistical tests were conducted at the significance level of $p<0.05$, and all reported p-values were two-tailed. We used Bonferroni correction to adjust for multiple comparisons when applicable.

References

1. Levine, R.L.; Garland, D.; Oliver, C.N.; Amici, A.; Climent, I.; Lenz, A.-G.; Ahn, B.-W.; Shaltiel, S.; Stadtman, E.R. Determination of Carbonyl Content in Oxidatively Modified Proteins. In Oxygen Radicals and Biological Systems Part B; Academic Press: New York, **1990**; pp. 464–478.

2. Karatepe, M. Simultaneous Determination of Ascorbic Acid and Free Malondialdehyde in Human Serum by HPLC-UV. LC-GC North Am. **2004**, 22, 362–365.

3. Mescam, M.; Vinnakota, K.C.; Beard, D.A. Identification of the Catalytic Mechanism and Estimation of Kinetic Parameters for Fumarase. *J. Biol. Chem.* **2011**, *286*, 21100–21109. https://doi.org/10.1074/jbc.M111.232033

4. Taniguchi, N.; Gutteridge, J. Experimental Protocols for Reactive Oxygen and Nitrogen Species, 1st ed.; Oxford University Press: New York, **2000**.

5. Li, Y.; Schellhorn, H.E. Rapid Kinetic Microassay for Catalase Activity. *J. Biomol. Tech.* **2007**, *18*, 185–187.

6. Grisham, M.B.; Johnson, G.G.; Lancaster, J.R. Quantitation of Nitrate and Nitrite in Extracellular Fluids. In Nitric Oxide Part A Sources Detect. NO; NO Synthase; Academic Press: New York, **1996**; pp. 237–246.

7. Rahman, I.; Kode, A.; Biswas, S.K. Assay for Quantitative Determination of Glutathione and Glutathione Disulfide Levels Using Enzymatic Recycling Method. *Nat. Protoc.* **2006**, *1*, 3159–3165. https://doi.org/10.1038/nprot.2006.378

Table S1. Pairwise PERMANOVA test among the samples of oxidative damage markers measured in the heart, liver, and kidney of nectarivorous, frugivorous, insectivorous, and hematophagous bats. Bolded numbers indicate statistically significant p-values (p<0.05).

		Heart				Liver				Kidney			
		Nectarivorous	Frugivorous	Insectivorous	Hematophagous	Nectarivorous	Frugivorous	Insectivorous	Hematophagous	Nectarivorous	Frugivorous	Insectivorous	Hematophagous
Carbonyl Groups	Nectarivorous		0.2511	0.5365	**0.0141**		1	**0.0072**	**0.0092**		**0.0038**	**0.0016**	**0.0024**
	Frugivorous			0.0636	**0.0237**			**0.0094**	**0.0072**			**0.0232**	**0.0054**
	Insectivorous				**0.0024**				**0.0002**				0.3972
	Hematophagous												
Malondialdehyde	Nectarivorous		1	**0.0374**	**0.0024**		**0.0038**	**0.0024**	**0.0024**		0.2299	1	0.6741
	Frugivorous			**0.0061**	**0.0016**			0.4503	**0.0038**			0.2299	**0.0107**
	Insectivorous				**0.0016**				**0.0024**				1
	Hematophagous												

Table S2. Pairwise PERMANOVA test among the samples of antioxidant enzymes measured in the heart, liver, and kidney of nectarivorous, frugivorous, insectivorous, and hematophagous bats. Bolded numbers indicate statistically significant p-values (p<0.05).

		Heart				Liver				Kidney			
		Nectarivorous	Frugivorous	Insectivorous	Hematophagous	Nectarivorous	Frugivorous	Insectivorous	Hematophagous	Nectarivorous	Frugivorous	Insectivorous	Hematophagous
H2O2 Consumption	Nectarivorous		0.2727	0.3301	**0.0038**		0.2364	**0.0199**	0.8598		1	**0.0061**	**0.0087**
	Frugivorous			1	**0.0026**			**0.0211**	0.2364			**0.0024**	1
	Insectivorous				**0.0037**				0.0551				**0.0038**
	Hematophagous												
Superoxide dismutase	Nectarivorous		0.2258	0.1035	**0.0016**		0.3229	0.1035	**0.0026**		**0.0374**	**0.0024**	0.5604
	Frugivorous			1	**0.0016**			1	**0.0026**			**0.0042**	**0.0042**
	Insectivorous				**0.0016**				**0.0026**				**0.0024**
	Hematophagous												
Fumarase	Nectarivorous		**0.0052**	**0.0374**	0.6013		**0.0038**	**0.0024**	**0.0038**		0.6718	**0.0024**	**0.0016**
	Frugivorous			1	**0.0056**			**0.0054**	**0.0116**			**0.0024**	**0.0016**
	Insectivorous				**0.0038**				0.815				**0.0224**
	Hematophagous												
Glutathione peroxidase	Nectarivorous		**0.0038**	1	**0.0056**		**0.0488**	**0.0024**	**0.0038**		0.1424	**0.0038**	0.5532
	Frugivorous			**0.0016**	1			1	**0.0056**			**0.016**	1
	Insectivorous				**0.0026**				**0.0038**				**0.0024**
	Hematophagous												
Glutathione-S-transferase	Nectarivorous		**0.0062**	**0.0047**	**0.002474**		**0.0088**	**0.0016**	**0.0038**		**0.0024**	0.7999	**0.0016**
	Frugivorous			**0.0283**	**0.002474**			0.0961	**0.0038**			**0.0371**	**0.0016**
	Insectivorous				**0.002474**				**0.0026**				**0.0056**
	Hematophagous												

Table S3. Pairwise PERMANOVA test among the samples of non-enzymatic antioxidants measured in the heart, liver, and kidney of nectarivorous, frugivorous, insectivorous, and hematophagous bats. Bolded numbers indicate statistically significant p-values (p<0.05).

		Heart				Liver				Kidney			
		Nectarivorous	Frugivorous	Insectivorous	Hematophagous	Nectarivorous	Frugivorous	Insectivorous	Hematophagous	Nectarivorous	Frugivorous	Insectivorous	Hematophagous
Total Glutathione	Nectarivorous		0.0842	0.2912	**0.0056**		1	1	**0.0201**		**0.0038**	**0.0026**	**0.0038**
	Frugivorous			1	**0.0038**			1	0.4982			**0.0016**	**0.0214**
	Insectivorous				**0.0038**				**0.0346**				**0.0016**
	Hematophagous												
Oxidazed Glutathione	Nectarivorous		1	0.5532	**0.0146**		0.8881	1	**0.0129**		0.0961	**0.0024**	1
	Frugivorous			**0.0119**	**0.0283**			1	**0.0392**			0.0606	0.0872
	Insectivorous				**0.0024**				**0.0437**				**0.0038**
	Hematophagous												
Reduced Glutathione	Nectarivorous		**0.0365**	0.6214	**0.0056**		0.0553	0.1442	0.9862		**0.0038**	**0.0038**	**0.0038**
	Frugivorous			1	**0.0038**			1	0.0891			**0.0034**	**0.0371**
	Insectivorous				**0.0056**				0.0891				**0.0024**
	Hematophagous												
GSSG/GSH	Nectarivorous		0.6697	0.1609	**0.0144**		0.3933	**0.0486**	**0.0486**		**0.0038**	**0.0056**	**0.0056**
	Frugivorous			1	**0.0271**			1	1			1	1
	Insectivorous				0.2571				1				0.1437
	Hematophagous												
Nitrites and Nitrates	Nectarivorous		0.0626	1	**0.0024**		**0.0033**	0.2961	**0.0064**		0.7999	1	**0.0271**
	Frugivorous			0.07658	**0.0065**			**0.0034**	1			0.3821	**0.0146**
	Insectivorous				**0.0016**				**0.0031**				**0.0488**
	Hematophagous												

Table S4. Pairwise PERMANOVA test among the samples of heart grouped according to bat species. Bolded numbers indicate statistically significant p-values (p<0.05).

	Nectarivorous	Frugivorous	Insectivorous	Hematophagous
Nectarivorous		**0.0257**	**0.021**	**0.025**
Frugivorous			**0.013**	0.889
Insectivorous				**0.023**
Hematophagous				

Table S5. Pairwise PERMANOVA test among the samples of liver grouped according to bat species. Bolded numbers indicate statistically significant p-values (p<0.05).

	Nectarivorous	Frugivorous	Insectivorous	Hematophagous
Nectarivorous		0.1041	0.7568	0.1041
Frugivorous			0.0640	**0.0489**
Insectivorous				**0.0451**
Hematophagous				

Table S6. Pairwise PERMANOVA test among the samples of kidney grouped according to bat species. Bolded numbers indicate statistically significant p-values (p<0.05).

	Nectarivorous	Frugivorous	Insectivorous	Hematophagous
Nectarivorous		**0.1041**	**0.0156**	**0.0104**
Frugivorous			**0.0140**	0.4041
Insectivorous				**0.0151**
Hematophagous				

Table S7. P-values for the Spearman's correlations between oxidative markers and (enzymatic and non-enzymatic) antioxidants measured in the heart. Bolded numbers indicate statistically significant p-values (p<0.05).

	Carbonyl	MDA	NO_2&NO_3	GSSG/GSH	VitC	H_2O_2↓	SOD	Fumarase	GPx	GST
Carbonyl		**0.0187**	**5.67E-08**	**0.0085**	**0.0401**	**0.0220**	**0.0043**	0.0688	**0.0192**	**0.0146**
MDA			**0.0012**	**3.62E-05**	**0.0001**	**1.18E-07**	**7.78E-08**	**0.0011**	0.4202	**9.18E-11**
NO_2&NO_3				**0.0040**	**0.0002**	**0,0001**	**2.18E-05**	**0.0148**	**2.42E-05**	**1.61E-05**
GSSG/GSH					**0.0037**	**0.0057**	**2.64E-05**	0.9933	0.3711	**1.24E-06**
VitC						**0.0006**	**1.01E-07**	0.4411	**0.0002**	**3.26E-06**
H_2O_2↓							**1.55E-05**	0.0878	**0.0362**	**2.55E-09**
SOD								**0.0229**	**0.0044**	**5.18E-09**
Fumarase									0.2907	8.86E-02
GPx										0.1304
GST										

Table S8. P-values for the Spearman's correlations between oxidative markers and (enzymatic and non-enzymatic) antioxidants measured in the liver. Bolded numbers indicate statistically significant p-values (p<0.05).

	Carbonyl	MDA	NO_2&NO_3	GSSG/GSH	VitC	H_2O_2↓	SOD	Fumarase	GPx	GST
Carbonyl		**0.0204**	**0.0117**	0.9723	**0.0252**	**0.0366**	**0.0377**	0.2912	**0.0007**	0.1373
MDA			**2.17E-06**	**0.0119**	0.6960	0.6951	**7.19E-06**	**0.0012**	0.1637	**1.46E-08**
NO_2&NO_3				0.2092	0.5923	0.6264	**0.0248**	0.2580	0.7547	**0.0020**
GSSG/GSH					0.2404	0.2150	0.1482	**0.0081**	0.4125	0.0636
VitC						0.2375	0.6589	0.1381	**1.04E-05**	0.9823
H_2O_2↓							0.8827	0.0860	0.0564	0.1557
SOD								**0.0001**	**8.53E-04**	**6.72E-10**
Fumarase									0.9797	**4.68E-06**
GPx										0.1152
GST										

Table S9. P-values for the Spearman's correlations between oxidative markers and (enzymatic and non-enzymatic) antioxidants measured in the kidney. Bolded numbers indicate statistically significant p-values ($p<0.05$).

	Carbonyl	MDA	NO_2&NO_3	GSSG/GSH	VitC	$H_2O_2\downarrow$	SOD	Fumarase	GPx	GST
Carbonyl		**0.0315**	0.0707	**0.0183**	0.2327	**0.0007**	0.3578	**0.0397**	**0.0251**	**0.0001**
MDA			**0.0084**	0.5599	0.2447	0.2957	0.4451	**0.0097**	0.6000	**0.0003**
NO_2&NO_3				0.8016	0.6027	0.3119	**0.0443**	0.0564	0.1844	**0.0005**
GSSG/GSH					**0.0064**	**0.0230**	**0.0092**	**0.0144**	**0.0099**	0.4437
VitC						0.2071	0.4458	0.4662	0.5592	0.9333
$H_2O_2\downarrow$							**0.0002**	**0.0092**	**0.0710**	0.1071
SOD								0.0781	**0.0211**	0.1766
Fumarase									**0.0074**	**0.0081**
GPx										0.9301
GST										

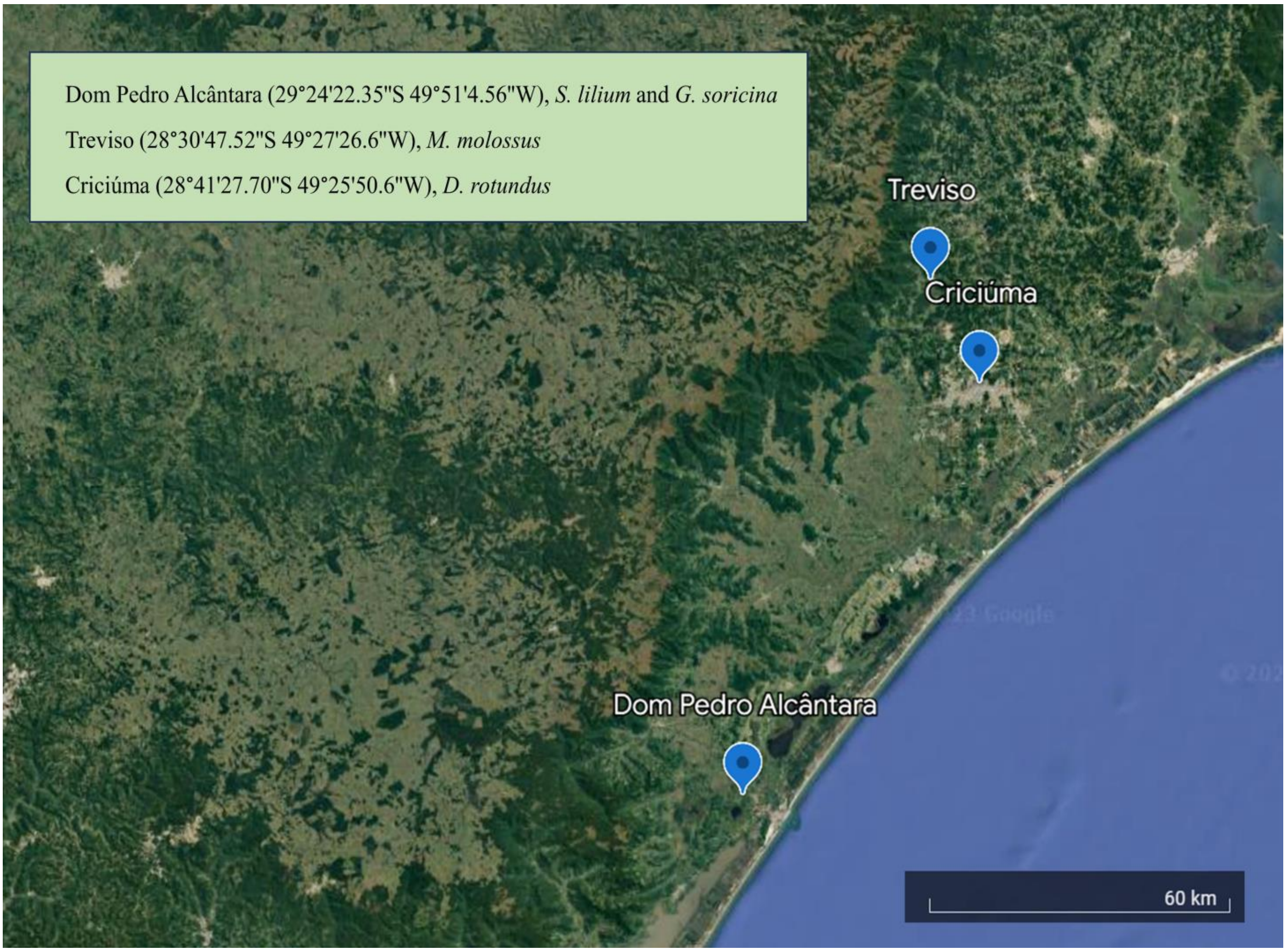

Figure S1 - Map of the collection sites and their coordinates.

Disclaimer/Publisher's Note: The statements, opinions and data contained in all publications are solely those of the individual author(s) and contributor(s) and not of MDPI and/or the editor(s). MDPI and/or the editor(s) disclaim responsibility for any injury to people or property resulting from any ideas, methods, instructions or products referred to in the content.
